# Rapidly progressive sarcomatoid malignant mesothelioma of the pleura mimicking pulmonary empyema

**DOI:** 10.1002/ccr3.371

**Published:** 2015-09-07

**Authors:** Kohei Fujita, Young Hak Kim, Koichi Nakatani, Tadashi Mio

**Affiliations:** 1 Division of Respiratory Medicine, National Hospital Organisation Kyoto Medical CentreKyoto, Japan; 2 Department of Respiratory Medicine, Graduate School of Medicine, Kyoto UniversityKyoto, Japan

**Keywords:** Empyema, malignant disease, mesothelioma, positron emission tomography–computed tomography

## Abstract

Refractory empyema occasionally reflects hidden malignant disease. We presented a rare case of rapidly progressive malignant mesothelioma of the pleura (MPM) mimicking empyema. Physicians should be aware of MPM when patients with empyema are refractory to the standard treatment, and PET-CT may be helpful in establishing a precise diagnosis in such cases.

## Introduction

Malignant pleural mesothelioma (MPM) has one of the poorest prognosis among respiratory diseases. Although the age-standardized incidence rates in males and females during 1999–2001 were only 12.5 to 3 per 100,000 person-years in Japan, respectively, MPM is still a major public health problem, as it is related to environmental and occupational asbestos exposure 1,2. An internationally conducted epidemiological survey estimated that the incidence peak of MPM in European countries will be reached between 2015 and 2020 3. To diagnose MPM, thoracoscopic biopsy is often needed; however, it is difficult to perform such an invasive procedure in patients of unstable clinical condition. Additionally, the clinical manifestations of MPM are usually nonspecific. Therefore, MPM is likely to be misdiagnosed as either tuberculous pleurisy or metastatic pleural lung cancer 4. Here, we present the case of a patient diagnosed and treated for pulmonary empyema, but was instead revealed by autopsy to have sarcomatoid MPM.

## Case Report

**Figure 1:**
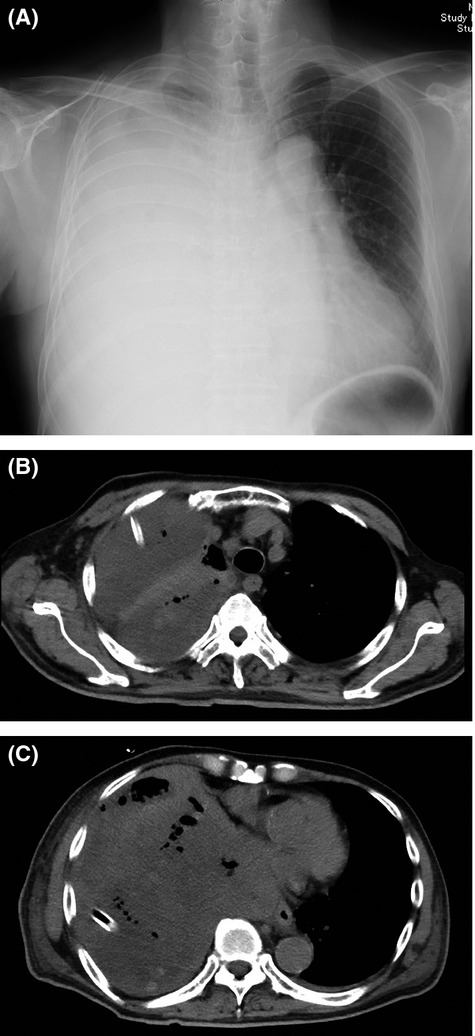
Chest radiography (A) at initial visit showed a massive pleural effusion on the right thorax. A Loculated pleural effusion was confirmed on the upper right thorax. Chest computed tomography (B, C) performed 2 weeks after admission confirmed massive enlarging loculated effusions. Two chest drainage tubes were separately inserted into the upper and lower right thorax.

**Figure 2:**
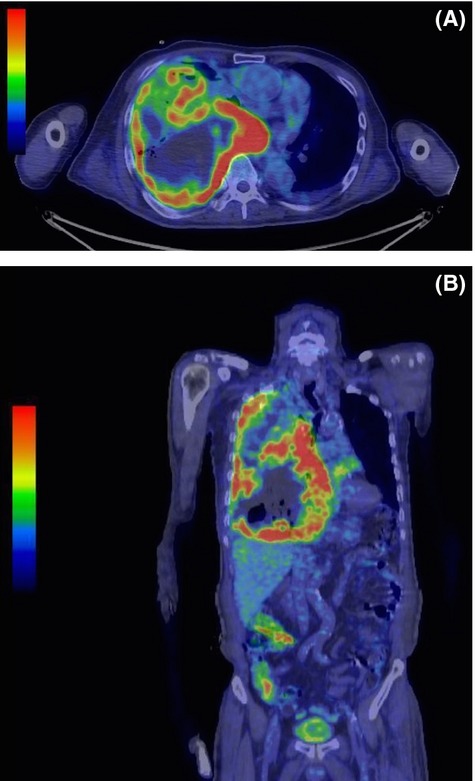
A positron emission tomography–computed tomography scan (A, B) revealed a high integration of fluorodeoxyglucose along the entire circumference of the pleura.

An 82-year-old man presenting with prolonged cough and dyspnea visited our hospital. Chest radiography (Fig.1A) revealed a massive loculated pleural effusion in right thorax. Neither pleural thickening nor pleural plaque was revealed following a computed tomography (CT) scan of the chest. The blood examination indicated a slight elevation of white blood cells, measured at 13,600/µL; C-reactive protein (CRP) was measured at 14.8 mg/dL, and lactate dehydrogenase (LDH) at 289 IU/L. Tumor markers were within normal levels (carcinoembryonic antigen [CEA] at 2.7 ng/mL, squamous cell carcinoma-related antigen at 1.3 ng/mL, and sialyl Lewis-x antigen at 27.5 IU/mL). We conducted an interferon-gamma-release assay, and it was negative. A chest drainage tube was immediately placed into the right thorax, and a total of 1200 mL of purulent pleural effusion was released. The specific gravity, LDH, and glucose levels of the pleural effusion were 1.029, 2140 IU/L, and 118 mg/dL, respectively. Neither adenosine deaminase (24.4 U/L) nor hyaluronic acid (43,800 ng/mL) was found to be elevated. No pathogens were cultured from the pleural effusion, and no malignant cells were detected. He had no obvious history of environmental or occupational asbestos exposure. A species of *Corynebacterium* was cultured from his pleural effusion. His initial diagnosis was pulmonary empyema. In addition to the drainage, an empiric course of antibiotic treatment with ampicillin/sulbactam was administered. During the first week, his clinical symptoms improved; however, two weeks after initial therapy, he developed an inflammatory reaction and high fever, and his condition declined rapidly. A chest CT scan revealed a remaining and enlarging loculated pleural effusion (Fig.1B and C). Another chest drainage tube was placed in the upper right thorax, but sufficient drainage was not achieved because of the hyperviscosity of the pleural effusion. The resistance of his condition to treatment with antibiotics and drainage therapy led us to consider the possibility of a malignant disease. A thoracoscopic lung/pleural biopsy was not performed because of his unstable vital signs. Although empyema with midline shift on chest radiography also suggested malignant disease, repeated cytologic analyses of his pleural effusion indicated no evidence of malignancy and elevation of CEA was absent in both serum and pleural effusion. As an alternative, positron emission tomography–computed tomography (PET-CT) was conducted, and intensive fluorodeoxyglucose (FDG) accumulation was detected throughout his entire right pleura (Fig.2A and B). These findings strongly suggested the presence of malignant mesothelioma.

A further examination was not performed due to his severe respiratory failure. He died one month after his initial visit to our hospital.

## Outcome and follow-up

**Figure 3:**
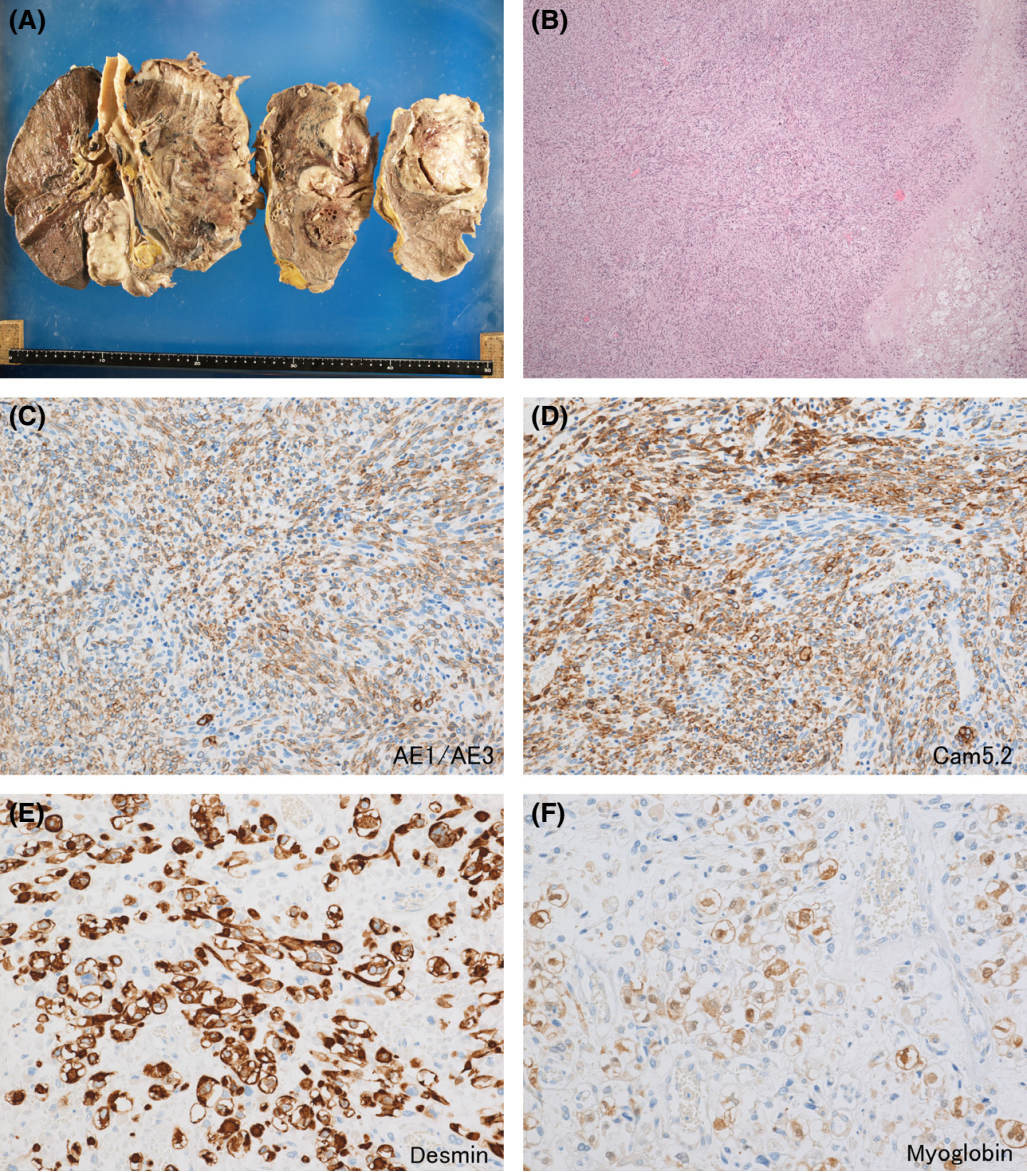
Autopsy specimens revealed large regions of necrotic tissue throughout the right lung (A). The dissected surface of the right lung indicated a massive progression of tumors. Histopathological analysis revealed a dense tumor characterized by spindle-shaped cells with atypical nuclei (B, H-E stain, ×100). Immunohistopathologic analysis demonstrated positive staining for AE1/AE3 (C, ×400) and for Cam5.2 (D, ×400). Focal positive staining for desmin (E, ×400) and myoglobin (F, ×400) was also observed.

An autopsy revealed large regions of necrotic tissue throughout the entire right thorax (Fig.3A). Dissecting the surface of his right lung revealed a massive progression of tumors. Histopathologic analysis demonstrated a dense tumor tissue characterized by spindle-shaped cells with atypical nuclei (Fig.3B). Immunohistopathology revealed extensive positivity for AE1/AE3 and CAM2.5 (Fig.3C and D). The focal regions of positivity for desmin and myoglobin were also revealed (Fig.3E and F), which indicated localized ectopic rhabdomyosarcoma. These findings comprehensively suggested that the patient suffered from sarcomatoid MPM.

## Discussion

Here, we presented a case of sarcomatoid MPM that was characterized by several unique and interesting features. First, the present case was mimicking pulmonary empyema. The pleural effusion associated with MPM usually has diverse properties, such as the presence of exudative or blood effusion, but it typically does not contain pus. Multilocular effusions are typically more indicative of empyema rather than MPM. Furthermore, our case lacked characteristic radiologic findings of MPM, such as pleural thickening and pleural plaque 5.

Second, the disease progression of the patient was quite rapid. MPM can be divided into three histologic types: the epithelioid type, sarcomatoid type, and mixed type 6. The sarcomatoid type is reported to encompass 4.9% to 25% of MPM cases 2,7. MPM usually progresses slowly over several decades following exposure to asbestos. MPM is a progressive disease, and the median survival time is approximately 10 months 2; however, extremely rapid progression of the disease, such as in the case presented, is uncommon.

A case report similar to ours has been previously published 8. Both ours and this previous case were initially treated as an empyema due to the presence of characteristic clinical findings, but both cases were refractory to the standard therapy that is used for empyema. In the previous case, a thoracoscopic examination was performed; however, the procedure focused entirely on the dissection of the empyema, and a histologic examination was not concurrently conducted. The diagnosis of sarcomatoid MPM was made over a course of months; by contrast, thoracoscopy was not performed in our case due to the unstable vital signs of the patient. As an alternative, PET-CT was performed in our case, and the intensive FDG accumulation throughout the patient's entire right pleura suggested the diagnosis of MPM. It has been reported that PET-CT is a sensitive method for identifying MPM 9. It has also been reported that the presence of high FDG uptake in the pleura in conjunction with scant FDG uptake in the surrounding empyema is a distinct sign of pleural malignant disease 10,11. When we are faced with a patient presenting with refractory empyema, we should consider the possibility that the patient might have MPM; further investigation of patient's condition using PET-CT may also be warranted.

## Conclusion

We presented a rare case of rapidly progressive sarcomatoid MPM mimicking pulmonary empyema. Physicians should be aware of MPM when patients with empyema are refractory to the standard treatment, and PET-CT may be helpful in establishing a precise diagnosis in such cases.

## References

[b1] Kanazawa N, Ioka A, Tsukuma H, Ajiki W, Oshima A (2006). Incidence and survival of mesothelioma in Osaka, Japan. Jpn. J. Clin. Oncol.

[b2] Nojiri S, Gemba K, Aoe K, Kato K, Yamaguchi T, Sato T (2011). Survival and prognostic factors in malignant pleural mesothelioma: a retrospective study of 314 patients in the west part of Japan. Jpn. J. Clin. Oncol.

[b3] Robinson BW, Lake RA (2005). Advances in malignant mesothelioma. N. Engl. J. Med.

[b4] Scherpereel A, Astoul P, Baas P, Berghmans T, Clayson H, de Vuyst P (2010). Guidelines of the European Respiratory Society and the European Society of Thoracic Surgeons for the management of malignant pleural mesothelioma. Eur. Respir. J.

[b5] Pairon JC, Andujar P, Rinaldo M, Ameille J, Brochard P, Chamming's S (2014). Asbestos exposure, pleural plaques, and the risk of death from lung cancer. Am. J. Respir. Crit. Care Med.

[b6] Attanoos RL, Gibbs AR (1997). Pathology of malignant mesothelioma. Histopathology.

[b7] Tanrikulu AC, Abakay A, Kaplan MA, Kucukoner M, Palanci Y, Evliyaoglu O (2010). A clinical, radiographic and laboratory evaluation of prognostic factors in 363 patients with malignant pleural mesothelioma. Respiration.

[b8] Matsuoka K (2014). Malignant pleural mesothelioma presenting as acute empyema with severe leukocytosis. Ann. Thorac. Cardiovasc. Surg.

[b9] Benard F, Sterman D, Smith RJ, Kaiser LR, Albelda SM, Alavi A (1998). Metabolic imaging of malignant pleural mesothelioma with fluorodeoxyglucose positron emission tomography. Chest.

[b10] Oh JK, Ahn MI, Kim CH, Cho KD, Cho DG, Kang CU (2008). The value of F-18 FDG-PET/CT in diagnosis of chronic empyema-associated malignancy. Clin. Radiol.

[b11] Haroon A, Zumla A, Bomanji J (2012). Role of fluorine 18 fluorodeoxyglucose positron emission tomography-computed tomography in focal and generalized infectious and inflammatory disorders. Clin. Infect. Dis.

